# Micronutrient and antinutrient content of semi-processed fruit peels: Towards boosting immunity

**DOI:** 10.4102/hsag.v29i0.2682

**Published:** 2024-08-29

**Authors:** Eridiong O. Onyenweaku, Hema Kesa

**Affiliations:** 1 Food Evolution Research Laboratory, School of Tourism and Hospitality, College of Business and Economics, University of Johannesburg, Johannesburg, South Africa

**Keywords:** micronutrients, antinutrients, fruit peels, dietary additives, immunity

## Abstract

**Background:**

Fruits are excellent sources of micronutrients; hence, their consumption is highly recommended. However, fruit peels, often discarded, despite some being edible have been reported to contain essential nutrients and antioxidants, which contribute to disease prevention and well-being.

**Aim:**

This study sought to evaluate the micronutrient and antinutrient content of 10 edible fruit peels namely, orange, mango, pineapple, banana, lemon, mandarin, red apple, cucumber, guava and pawpaw peels.

**Setting:**

Laboratory analyses of the fruit peels were conducted at the University of Calabar, in a well-ventilated and temperature controlled environment to ensure accurate results.

**Methods:**

The research design was quantitative and experimental; laboratory analyses were carried out to determine the minerals, vitamins and antinutrients in fruit peels using AOAC standard methods. Data were analysed using ANOVA on SPSS version 20.0.

**Results:**

Calcium was the most predominant of the minerals analysed, with values ranging from 33.12 ± 0.05 mg/100 g (cucumber peels) to 72.04 ± 0.08 mg/100 g (mango peels); calcium was followed by magnesium. Mandarin, banana and pineapple peels had statistically similar (*p* > 0.05) content of zinc (about 1.2 mg/100g), which was significantly (*p* < 0.05) higher than others. Mandarin peels had significantly higher content (100.48 ± 2.03 mg/100 g) of vitamin C, while cucumber peels recorded the lowest (27.50 ± 0.01 mg/100 g). The results show significant concentrations of micronutrients such as iron, selenium and vitamin K; among the antinutrients, hydrogen cyanide had the highest concentrations, followed by phytate. The values were within safe limits.

**Conclusion:**

Therefore, the processing of fruit peels, for use as dietary additives to enrich foods and boost immunity, should be promoted.

**Contribution:**

The study shows the potential of fruit peels as food additives.

## Introduction

Fruits are naturally a great source of vitamins, minerals, antioxidants and phytonutrients (Slavin & Lloyd 2012). The current rise in the prevalence of diseases and a concurrent drop in life expectancy has triggered studies (on natural remedies) trying to find solutions to the various health challenges that have come with industrialisation and globalisation. Antioxidants have been discovered to be useful in eliminating and diminishing the action of free radicals, which cause oxidative stress; they are able to achieve this via antioxidative defence mechanisms (Rajendran et al. 2014). Antioxidants are those substances that possess free radical chain reaction breaking properties. Many antioxidants are vitamins and minerals such as vitamin A, E, selenium and ascorbic acid. Micronutrients are essentially required by humans and other organisms in varying amounts throughout life to coordinate various physiological functions to maintain health (Tucker 2016). Micronutrients (vitamins and minerals) have numerous health benefits, including tissue maintenance, bone and teeth formation and health, serving as cofactors and coenzymes to enzyme various enzyme systems, aiding the regulation and coordination of most body functions and other biochemical and physiological functions in the body (Gernand et al. 2016) (Godswill et al. 2020). Thus, consumption of healthy diets that are rich in these micronutrients is important in the prevention and management of both chronic and infectious diseases.

The World Health Organization (2020) has recommended consuming 400 g of fruit per day at a minimum, citing evidence that larger fruit intakes may protect against some malignancies and cardiovascular disorders. This recommendation has resulted in the implementation of the ‘5-a-day’ fruit and vegetable campaign in numerous countries (Oyebode et al. 2014), leading to an increase in the consumption of fresh fruits. However, because of the seasonal nature and limited shelf-life of most fruits, a large portion of the fruit supply is processed to prolong availability throughout the year. During this processing, the peels of most fruits are usually taken off and discarded as waste in the food industry. Fruit peel serves as a rich source of dietary fibre, also referred to as non-soluble polysaccharides (NSPs), including hemi-cellulose, pectin, tannins and gum, among others (Dhiman, Ahmad & Nanda 2011). These compounds enhance the bulk of food and aid in preventing constipation by reducing gastrointestinal transit time. Fruit peels are also low in energy value, sugar and fats and are devoid of cholesterol, thereby contributing to decreased overall food intake (Moon & Shibamoto 2009). Utilising fruit by-products, particularly peels, which can represent up to 30% of the total weight in some fruits, has gained traction, especially as research has revealed their superior biological activities compared to other fruit parts (Feumba, Ashwini & Ragu 2016; Moon & Shibamoto 2009). Moreover, with the growing interest in natural sources of bioactive compounds and the popularity of functional foods, there is a rise in the development of food products enriched with fruit peels (Altunkaya et al. 2013; Wisal, Abdel & Sirekhatim 2013).

Furthermore, rather than discarding fruit peels at both household and food industry levels, it would be beneficial to incorporate them as dietary additives to meals. This would then increase the nutritional value of those meals and reduce food waste. For example, these fruit peels could be dried and used as sprinkles (food additives) in meals, thereby enriching the diet and reducing food losses. Processed fruit peels have the potential to generate additional income among farmers and others involved in the processing of the fruit peels for commercial purposes. In order to ascertain how safe these fruit peels are for consumption, it is important to determine the antinutrient levels and, if high, seek processing methods that can reduce the antinutrient levels. This will ensure optimal bioavailability of the essential micronutrients present in the fruit peels. For example, an antinutrient such as oxalate binds calcium and reduces calcium bioavailability in the body (Salgado et al. 2023). This can lead to issues of calcium deficiency manifested as bone and teeth problems if nothing is done about it.

Summarily, this study sought to evaluate the micronutrient content (minerals and vitamins) and antinutrient content of ten different fruit peels, namely mango, cucumber, lemon, orange, pawpaw, banana, guava, red exotic apple, mandarin and pineapple peels. The second phase of this research will be aimed at processing the fruit peels into fruit sprinkles that can be used as dietary additives. The fruit peels will undergo the process of de-bittering and will be produced in different flavours (such as salty, spicy, sweet and bland) based on consumer preferences.

## Research methods and design

This research design was experimental and a quantitative approach was employed.

### Sample collection and preparation

About 3 kg each of fresh and matured samples of mango (*Mangifera indica*), cucumber (*Cucumis sativus*), lemon (*Citrus limon*), orange (*Citrus sinensis*), pawpaw (*Carica papaya*), banana (*Musa paradisiaca*), guava (*Psidium guajava*), red exotic apple (*Malus domestica*), mandarins (*Citrus reticulata*) and pineapple (*Ananas comosus*) fruits were collected from local South African markets. To ensure accurate results, extra care was applied in selecting fruits that were free from visible blemishes and bruises. The fruits were purchased at different stages of ripeness, with some being unripe and others being fully ripe (based on availability). The unripe fruits were allowed to naturally ripen at room temperature, while the ripe fruits were prepared immediately for analysis.

The sample preparation process involved sorting and then washing the fruits thoroughly under clean running water to remove any surface dirt, debris or other contaminants before draining. The fruits were then peeled carefully using a sharp peeler to ensure that no pulp remained on the peel. Finally, the fruit peels were air dried in a well-ventilated room to remove excess moisture and then ground to obtain a fine powder using a Binatone (BLG 620) blender at 3000 rpm. These steps were taken to ensure that the samples were in a consistent, dry powdered state for analysis and to minimise any potential variability in the results because of differences in moisture content or other factors.

### Laboratory analyses

#### Determination of sodium

Sodium was determined by flame photometry method as described by James (1995). The instrument (photometer) was set up; 5 mL of prepared sodium standard solution was aspirated into the machine and sprayed over the non-luminous butane gas flame. The sodium emission (having been appropriately filtered) from the different concentrations was recorded and made into the standard curve. Subsequently, the optical density emissions recorded from each of the samples were against those in the curve, using the curve to extrapolate the quality of sodium in the sample.

#### Determination of calcium

Calcium was determined using the method described by AOAC (1990); 25 mL of the digested sample was pipette into 250 mL conical flask and a pinch of Eriochrome Black-T-Indicator (EBT) was added. Thereafter, 2 mL of 0.1M NaOH solution was added, and the mixture was titrated with standard EDTA (0.01M EDTA) solution.

Calculation:


$$Calcium(mg/l)=\frac{T\times M\times E\times 100}{\text{Volume of sample used}}$$ [Eqn 1]

where:

*T* = Titre value

*M* = Molarity of EDTA

*E* = Equivalent weight of calcium.

#### Determination of potassium

Potassium was determined by a procedure described by AOAC (2005) using a flame photometer. Potassium standard was prepared and used to calibrate the instrument read out. The meter reading was at 100% E (emission) to aspire the top concentration of the standards. The % E of all the intermediate standard curves was plotted on linear graph paper with these readings. The concentration of the element in the sample solution was read from the standard curve.

Calculation:


$$\%Potassium=\frac{ppm\times 100\times DF}{1\;million}$$ [Eqn 2]

#### Determination of magnesium

The atomic absorption spectrophotometer (AAS) method, as described by Nnadi (2020), was used. Atomic absorption spectrophotometer was set up using magnesium (Mg) hollow cathode lamp at a wavelength of 202.6 nm. Magnesium standard solution of concentrations: 0.5, 1.0, 2.0 and 4.0 ppm were prepared from the standard stock solution. The equipment was ‘zeroed’ using de-ionized water. Magnesium standard was run to obtain the standard plot. The sample digest was run, and the values displayed on the screen were also recorded, and the magnesium content was calculated.

#### Determination of micro-elements

Micro-element content of the samples was determined using atomic spectrophotometric absorption method as described by AOAC (1990). Samples were first prepared by digestion using an acid mixture of nitric acid, perchloric and sulphuric acid. The digest was later filtered and diluted to 50 mL mark with distilled water. Preparation of standard solutions of the elements were prepared from the standard solution containing 1000 ppm of each element in 2N nitric acid solution. The calibration and measurement of each element against a blank was performed at different working wavelengths (see Table 1).

Calculation:


$$Element=\frac{Au}{\text{As}}\times ppm\;of\;curve\times \frac{Vf}{\text{Vx}}\times \frac{1000}{W}$$ [Eqn 3]

*A*_u_ = Absorbance of sample

*A*_s_ = Absorbance off standard

*V*_f_ = Total volume of extract

*V*_x_ = Total volume of extract used

*W* = Weight of sample used.

**TABLE 1:** Spectrophotometric wavelengths of various elements.

S\No	Elements	Wavelength (nm)
1	Zinc	213.8
2	Iron	234
3	Manganese	246
4	Selenium	384

S\No, serial number.

#### Determination of vitamin A

The AOAC (1990) method using the colorimeter was adopted. This measures the unstable colour at the absorbance of 620 nm that result from the reaction between vitamin A and SbL_3_.

Calculation:


$$Vitamin\;A\;(mg/100g)=A620nm\times SL\times \frac{V}{Wt}$$ [Eqn 4]

where:

*A*620 *nm* = Absorbance at 620 nm

*SL* = Slope of standard curve (Vit. A conc.) + A620 nm reading

*V* = Final volume in colorimeter tube

*Wt* = Weight of sample.

#### Determination of vitamin C

The sample was determined using the titrimetric method described by AOAC (2010); 5 g of sample was dispensed in 50 mL of EDTA/TCA solution and homogenised. The homogenate was filtered with Whatman no. 42 filter paper, and more of the extractant was used to wash the residue in the filter paper until 50 mL filtrate was obtained. A 20 mL portion of the filtrate was measured into a conical flask, and 10 mL of 30% potassium solution was added to it, mixed well and then followed by 1% starch solution. The mixture was titrated against 0.01 M CuSO_4_ solutions. A reagent blank was also titrated. The vitamin C content was calculated based on the relationship that 1 mL 0.01 M CuSO_4_ = 0.88 mg vitamin C:


$$Vitamin\;C\;(mg/100g)=\frac{100}{W}\times 0.88\times (T-B)\times \frac{Vt}{Va}$$ [Eqn 5]

*W* = Weight of sample

*T* = Titre value of sample

*B* = Titre valve of blank

*V_t_* = Total extract volume

*V_a_* = Volume of extract titrated.

#### Determination of vitamin E

Vitamin E was determined using the colorimetric method described by AOAC (2010). The absorbance was read in a spectrophotometer at 470 nm wavelength.

Calculation:


$$Vitamin\;E\;(mg/100g)=\frac{a-b\times c}{s-b\times w}$$ [Eqn 6]

where,

*a* = absorbance of test sample

*b* = absorbance of the standard solution

*c* = concentration of standard in mg/100g w = weight of the sample used.

#### Determination of vitamin K

This was determined using AOAC method (2010), the sample (1 g) was weighed, macerated with 20 mL of distilled H_2_O and filtered. The filtrate (1 mL) was pipetted into a test tube, and 1 mL of 0.04% 2, 4-dinitrophenylhydrazine in 20% HCl solution was added. It was then boiled for 45 mins and cooled. The solution was diluted to 10 mL with 1:30 ammonium hydroxide and the absorbance was measured at a wavelength of 635 nm.

Calculation:


$$Vitamin\;K\;(mg/100g)=\frac{Au}{As}\times Slope$$ [Eqn 7]

#### Determination of antinutrients

Tannin: This was determined using the colorimetric method described by AOAC (2005). Their respective absorbance was measured in a spectrophotometer at 260 nm using the reagent blank to calibrate the instrument at zero.

Phytate: Phytate content was determined using spectrophotometric method as described by AOAC (1975):


$$\text{Phytate }(\text{mg})=\frac{\text{concentration of phytate}(\text{mg}/100\text{g})\text{ from standard curve}\times \text{dilution factor}}{\text{Weight of sample}}$$ [Eqn 8]

Hydrogen Cyanide: This was determined by the alkaline picrate colorimetric method by AOAC (1975); the cyanide content (HCN) in mg/kg was calculated using the formula:


HCN (mg/kg)=(100/W)×Au (As) [Eqn 9]

where,

*W* = Weight of sample analysed (g)

*Au* = Absorbance of sample (nm)

*As* = Absorbance of the standard HCN solution (nm).

*Oxalate:* Oxalate was determined titrimetrically by using the method described by Olumuyiwa et al. (2003). After the procedure, the solution was then warmed to 70°C–80°C and titrated until a permanent pink colour that persisted for at least 30 s was attained. The oxalate content was calculated as sodium oxalate equivalent.

*Phenol:* The total phenol content was determined according to the method of Meda et al. (2005) using gallic acid as standard. Total phenolic content was expressed as gallic acid equivalents (the concentration of gallic acid was established from a calibration curve) in milligram per gram fresh weight (mg GAE/g).

### Statistical analysis

Laboratory results generated from this study were statistically analysed using the Statistical Package for Social Science (SPSS) version 20.0. One-way analysis of variance (ANOVA) was used to compare the nutrient values and check for significant differences among groups. All the results were expressed as mean ± SEM for 3 determinations, and statistical significance was accepted at *p* < 0.05 (95% confidence level).

### Ethical considerations

This article does not contain any studies involving direct human or animal contact. Ethical approval was received from the University of Johannesburg STH Research Ethics Committee with clearance code 21STH08.

## Results

### Mineral composition of the edible fruit peels

The macro and microelement composition of the ten edible fruit peels analysed in this study is reported in Table 2. Calcium had the highest concentrations among the minerals analysed, with values ranging from 33.12 ± 0.05 mg/100 g (cucumber peels) to 72.04 ± 0.08 mg/100 g (mango peels). Magnesium was the next most predominant macro element found in the samples, and the values ranged from 22.02 ± 0.01 mg/100 g (cucumber peels) to 31.77 ± 0.08 mg/100 g (red apple peels). Sodium concentrations varied from 10.01 ± 0.01 mg/100 g (cucumber peels) to 23.50 ± 0.05 mg/100 g (red apple peels). On the other hand, the potassium content of the fruit peels was quite low with banana peels having significantly (*p* < 0.05) higher content (3.64 ± 0.01 mg/100 g) than the others – cucumber peels recorded the least potassium content (0.09 mg/100 g). Mandarin, banana and pineapple peels had statistically similar (*p* > 0.05) content of zinc (about 1.2 mg/100 g), which was significantly (*p* < 0.05) higher than others. Pawpaw peels had a significantly higher concentration of iron (0.83 mg/100 g) than the other fruit peels, with cucumber peels recording the lowest iron concentration (0.36 mg/100 g).

**TABLE 2:** Mineral composition (mg/100 g) of the processed edible fruit peels.

Fruit peels	Na	Ca	K	Mg	Zn	Se	Fe	Mn
Mango	21.04	72.04	1.21	30.21	0.92	1.60	0.51	2.98
	± 0.04^b^	± 0.08^i^	± 0.01^b^	± 0.07^a^	± 0.05^a^	± 0.02^a^	± 0.00^a^	± 0.02^a^
Cucumber	10.01	33.12	0.09	22.02	0.81	1.52	0.36	3.06
	± 0.01^a^	± 0.04^a^	± 0.00^a^	± 0.04^a^	± 0.03^a^	± 0.02^a^	± 0.00^b^	± 0.01^a^
Lemon	11.14	62.22	2.16	25.41	0.73	1.80	0.71	2.81
	± 0.02^a^	± 0.10^f^	± 0.01^b^	± 0.03^a^	± 0.02^a^	± 0.05^a^	± 0.00^a^	± 0.01^a^
Orange	16.76	65.01	1.22	27.06	0.90	1.75	0.62	2.50
	± 0.01^b^	± 0.05^g^	± 0.01^b^	± 0.02^a^	± 0.02^a^	± 0.10^a^	± 0.01^c^	± 0.01^a^
Pawpaw	12.53	40.98	2.82	23.42	0.71	2.03	0.83	3.17
	± 0.02^b^	± 0.07^b^	± 0.01^b^	± 0.02^a^	± 0.01^a^	± 0.20^a^	± 0.00^a^	± 0.00^a^
Mandarins	20.49	51.97	2.47	28.72	1.22	1.64	0.69	3.44
	± 0.01^e^	± 0.52^e^	± 0.01^c^	± 0.06^c^	± 0.01^b^	± 0.01^d^	± 0.01^f^	± 0.02^d^
Pineapple	17.79	42.39	2.79	24.07	1.18	1.49	0.57	2.79
	± 0.03^c^	± 0.13^c^	± 0.02^d^	± 0.59^b^	± 0.01^b^	± 0.01^c^	± 0.01^d^	± 0.07^c^
Banana	22.69	49.50	3.64	25.68	1.26	2.32	0.64	2.29
	± 0.05^f^	± 0.00^d^	± 0.01^f^	± 0.12b^c^	± 0.01^b^	± 0.01^e^	± 0.01^e^	± 0.02^e^
Guava	19.35	41.29	3.40	22.78	0.96	1.65	0.78	4.65
	± 0.02^d^	± 0.01^b^	± 0.00^e^	± 0.02^b^	± 0.33^b^	± 0.01^d^	± 0.00^h^	± 0.07^d^
Red apple	23.50	68.52	2.83	31.77	0.87	1.40	0.73	3.72
	± 0.05^g^	± 0.32^h^	± 0.80^e^	± 0.08^d^	± 0.37^b^	± 0.01^b^	± 0.01^g^	± 0.15^b^

Note: Values are expressed as mean ± SEM, *n* = 3. Values in the same column with similar superscripts (a-h) are statistically similar at *p* < 0.05.

Na, sodium; K, potassium; Ca, calcium; Mg, magnesium; Z, zinc; Se, selenium; Fe, iron; Mn, manganese.

### Vitamin content of the edible fruit peels

Table 3 shows the vitamin composition (both fat-soluble and water-soluble vitamins) of the edible fruit peels. The vitamins analysed were chosen based on a literature review, which gave an idea of the vitamins that would be found in significant quantities in this type of food material. Mandarin peels had significantly (*p* < 0.05) higher content (100.48 ± 2.03 mg/100 g) of vitamin C, while cucumber peels recorded the lowest (27.50 ± 0.01 mg/100 g). The fruit peels showed considerable concentrations of vitamin E as well, with values ranging from 2.50 ± 0.75 mg/100 g (red apple peel) to 8.10 ± 0.02 mg/100 g (cucumber peel). Pawpaw peel had significantly (*p* < 0.05) higher content of vitamin K (14.70 ± 0.03 mg/100 g), which differed from the others; mango peel had the lowest concentration of vitamin K (1.97 ± 0.01 mg/100 g), which was significantly (*p* < 0.05) different from the other sample values.

**TABLE 3:** Vitamin composition of the edible fruit peels (in mg/100 g).

Fruit peels	B-carotene	Vit. C	Vit. E	Vit. K
Mango	2.97 ± 0.03^a^	63.26 ± 1.63^d^	6.71 ± 0.03^d^	1.97 ± 0.01^a^
Cucumber	4.74 ± 0.02^b^	27.50 ± 1.44^a^	8.10 ± 0.02^e^	2.36 ± 0.03^b^
Lemon	2.08 ± 0.01^a^	27.67 ± 1.45^a^	6.82 ± 0.02^d^	3.61 ± 0.01^c^
Orange	1.93 ± 0.02^a^	47.67 ± 1.45^b^	8.02 ± 0.03^e^	4.20 ± 0.01^d^
Pawpaw	5.12 ± 0.03^c^	32.50 ± 1.44^a^	5.34 ± 0.02^c^	4.70 ± 0.03^e^
Mandarins	4.89 ± 0.02^b^	100.48 ± 2.03^g^	3.63 ± 0.02^b^	2.47 ± 0.01^b^
Pineapple	6.74 ± 0.01^d^	56.05 ± 1.42^c^	4.83 ± 0.02^c^	2.78 ± 0.04^b^
Banana	6.92 ± 0.01^d^	68.20 ± 1.71^e^	4.94 ± 0.01^c^	3.64 ± 0.02^c^
Guava	4.33 ± 0.02^b^	95.04 ± 1.95^f^	3.47 ± 0.01^b^	3.40 ± 0.00^c^
Red apple	2.81 ± 0.91^a^	61.40 ± 2.01^d^	2.50 ± 0.74^a^	3.34 ± 0.08^c^

Note: Values are expressed as mean ± SEM, *n* = 10. Values in the same column with different superscripts (a-g) are significantly different (*p* < 0.05).

Vit. C, vitamin C; Vit. E, vitamin E; Vit. K, vitamin K.

### Antinutrient content of the edible fruit peels

Results of the antinutrients analyses of the ten edible fruit peels are shown in Table 4. Hydrogen cyanide was the most predominant among the five nutrients analysed in this study with values ranging from 4.86 ± 0.01 mg/100 g in guava peel to 18.72 ± 0.15 mg/100 g in pawpaw peel. No significant content of tannin was found in cucumber, lemon, orange and pawpaw peel, while mandarin peels recorded the highest tannin value (1.93 ± 0.01 mg/100 g). Guava peel had the highest oxalate concentration (2.87 ± 0.02 mg/100 g), which was significantly (*p* < 0.05) higher than the others; cucumber peel had the lowest oxalate content of 0.29 ± 0.01 mg/100 g, which also significantly (*p* < 0.05) differed from the others. The concentrations of phytate in the fruit peels ranged from 1.55 ± 0.01 in banana peel to 5.89 ± 0.02 mg/100 g in pawpaw peel.

**TABLE 4:** Antinutrient content of the powdered fruit peels (mg/100 g).

Fruit peels	Tannins	Oxalates	Phytates	Phenols	Hydrogen cyanide
Mango	0.01	0.46	4.51	2.40	7.40
	± 0.00^a^	± 0.01^c^	± 0.01^e^	± 0.03^e^	± 0.01^c^
Cucumber	0.00	0.29	6.75	0.42	12.09
	± 0.00^a^	± 0.01^a^	± 0.13^g^	± 0.02^b^	± 0.05^d^
Lemon	0.00	0.61	4.52	2.60	12.49
	± 0.00^a^	± 0.01^e^	± 0.01^e^	± 0.04^f^	± 0.07^e^
Orange	0.00	0.38	4.52	1.16	7.59
	± 0.00^a^	± 0.01^b^	± 0.01^e^	± 0.01^c^	± 0.05^c^
Pawpaw	0.00	0.53	5.89	1.23	18.72
	± 0.00^a^	± 0.02^d^	± 0.07^f^	± 0.01^d^	± 0.15^f^
Mandarins	1.93	2.86	2.57	0.61	8.73
	± 0.01^d^	± 0.02^i^	± 0.01^b^	± 0.01^a^	± 0.01^b^
Pineapple	1.86	2.61	1.69	0.45	9.83
	± 0.00^c^	± 0.01^h^	± 0.00^a^	± 0.00^a^	± 0.02^b^
Banana	1.77	1.93	1.55	0.39	6.42
	± 0.01^b^	± 0.01^f^	± 0.01^a^	± 0.01^a^	± 0.01^a^
Guava	1.92	2.87	3.85	0.55	4.86
	± 0.01^d^	± 0.02^i^	± 0.01^d^	± 0.01^a^	± 0.01^b^
Red apple	1.83	2.45	2.77	0.43	8.70
	± 0.02^c^	± 0.01^g^	± 0.01^c^	± 0.00^a^	± 0.04^b^

Note: Values are expressed as mean ± SEM, *n* = 10. Values in the same column with different superscripts (a-i) are significantly different (*p* < 0.05).

## Discussion

The results of this study also show that fruit peel is the potential source of vitamins, and a higher concentration was present in cucumber and mango fruit peels, although the minerals were present in considerable amounts. The findings of this research are also in line with the previous study of Feumba et al. (2016), who reported a high amount of vitamins and minerals in fruit peels. Minerals are essential for many physiological processes in the body, particularly those involving synthesis and regulation (Soetan et al. 2010). According to Ismail et al. (2014), fruits (including their various parts) are regarded as a good source of dietary minerals. The macroelements in this study – sodium, potassium, magnesium and calcium are very essential for physiological activities in the body including the regulation of blood pressure and formation of healthy bones and teeth. Calcium also plays a key role in controlling muscle and nerve activity (Feumba et al. 2016). Furthermore, it was observed that calcium values for the red exotic apple peels are similar to those obtained by Manzoor et al. (2012), showing that apple peels contain useful amounts of calcium as this would contribute a significant percentage of the recommended dietary allowance (RDA). Calcium content in mango, red apple and some other fruit peels reported in this study was slightly higher than those reported by Feumba et al. (2016), but they recorded higher calcium in orange peels. The results for sodium and potassium in this study corroborate the high potassium content in banana peels, as reported by Anhwange, Ugye and Nyiatagher (2009). According to Alfonzo et al. (2006), the increased availability of potassium could also help with the management of renal disruptions, breathing stress and cardiac malfunction. Magnesium values in this study were relatively higher than those recorded for similar but not exact fruit peels by Hussain, Kalhoro and Yin (2023); this could be caused by differences in fruit variety, level of ripeness and variations in analytical methods.

Trace elements, such as iron, selenium, manganese and zinc, are vital micronutrients that perform various biochemical roles in all life forms (Dhiman et al. 2011). Iron is essential for blood function, the creation of collagen, energy production and healthy immune system operation as it transports oxygen to the cells. Similar results indicating small concentrations of iron were reported by Hussain et al. (2023) for citrus, guava and red apple peels. Furthermore, the iron content in banana peels was relatively lower than that reported for banana, orange and pineapple peels by Feumba et al. (2016). These discrepancies could be affected by a number of factors such as fruit variety, nature of cultivating soil and weather conditions. In addition, zinc is very important for cellular reproduction and immune system development. Zinc also plays a crucial role in growth; it has been found to influence more than 300 enzymes via contributing in their structural, catalytic and regulatory functions (Feumba et al. 2016). The zinc content reported in this study for red apple peels and many others was comparable to that reported by similar studies showing considerable zinc content in fruit peels. However, zinc for mandarin peels reported in this study was slightly lower than that reported by Feumba et al. (2016) and Dhiman et al. (2011) for oranges in the citrus genus, which could be as a result of age disparity in fruit as zinc concentration in plants is influenced by the age and vegetative state of the plant with higher zinc concentration being found in younger plants (Dhiman et al. 2011). Another important element is selenium, a trace mineral that is vital to human health. In this study, selenium concentrations were low, but this is not a problem as the element is required in very small quantities by the body. It serves multiple functions, including acting as an antioxidant to protect cells from oxidative stress and chronic illnesses (Tóth & Csapó 2018). It also boosts the immune system and prevents diseases (Tóth & Csapó 2018). The recommended daily intake of selenium for humans is 55 micrograms (mcg), which the fruit peels can supply (Institute of Medicine 2000).

Looking at the vitamin content, the results prove that fruit peels can serve as a potential source of vitamin C and carotenoids and hence can be used in the management of micronutrient deficiencies such as scurvy and night blindness, and their consumption should be encouraged through incorporation into foods (Godswill et al. 2020). For instance, the peel of oranges contains more vitamin C (ascorbic acid) than the juice itself (Rafiq et al. 2018). Additionally, some fruit peels have been reported to serve as a rich source of vitamin A, B-complex vitamins and minerals such as calcium, selenium, manganese and zinc, often in higher concentrations than the pulp (Devi et al. 2023). The values for the vitamins recorded in this study were slightly similar to the concentrations reported by Hussain et al. (2023), while Hemalatha and Anbuselvi (2013) reported higher vitamin C values (26.5 mg/100 g) for pineapple wastes, which could be attributed to differences in varieties and components attached to the peels. The fruit peels were found to have significant amounts of vitamin E, which can contribute to the RDA of 15 mg per day. Vitamin E is known for its strong antioxidant properties, which help prevent chronic diseases and eliminate free radicals while suppressing lipid peroxidation (Sarkar et al. 2020). Vitamin K is a crucial nutrient for blood clotting, bone and blood vessel health and energy metabolism. The values for vitamin K in this study would make significant percentage contribution to the RDA for vitamin K (120 mcg per day).

The antinutrients in this study were found in minimal amounts, which were within tolerable limits as stipulated by the European Food Safety Authority (Aniemeka & Ndubuisi 2017). Although certain phenolic compounds such as flavonoids have protective effects including anti-inflammatory, anti-oxidant, anti-viral and anti-carcinogenic properties, at very high concentrations they exhibit a myriad of antinutrient activities; they can chelate metals such as iron, copper and zinc and decrease their absorption (Chukwuebuka & Chinenye 2015). They also inhibit digestive enzymes and may precipitate proteins (Enechi, Ugwu & Ugwu 2013). Oxalates can bind to calcium in food, thereby rendering calcium unavailable for normal physiological and biochemical roles (Feumba et al. 2016). Tannin was not found in considerable amounts in most of the fruit peels; the highest tannin content was in mandarin peels. This is slightly similar to the observations of Aina, Oyedeji and Adegboyega (2019), which had slightly higher tannin values in banana peels; this variation could arise from environmental influences, genetic factors and difference in methodology. Phenol concentrations were also quite low, but this is contrary to Ghasemi, Ghasemi and Ebrahimzadeh (2009), who reported that the total phenolic content in mandarin and some other citrus fruit peels ranged from 104 to 172 mg/100 g. This variation may be due to the geographical location, soil type and analytical method. On the other hand, phenols and polyphenolic compounds, such as flavonoids, are widely found in food products derived from plant sources, and they have been shown to possess significant antioxidant activities (Ghasemi et al. 2009). The correlation between total phenol contents and antioxidant activity has been widely studied in different foodstuffs such as fruits and vegetables (Klimczak et al. 2007; Kiselova et al. 2006) (Van Acker, Bast & Van Der Vijgh 2010). Hydrogen cyanide recorded the highest concentrations of antinutrients found in this study; this was far lower than the values reported by Anhwange et al. (2009) (133 mg/100 g) for HCN in banana peels. This could be because of variations in varieties and environmental factors.

Summarily, it has become necessary to develop other sustainable alternatives to meet the demand of the world’s expanding population, thereby meeting the sustainable development goals (SDGs) of poverty eradication, zero hunger and climate action. One possible technique is to use fruit peels as food additives and food fortifiers. These fruit peels have been found to have useful amounts of micronutrients such as magnesium, calcium, iron, zinc, citrate content and other minerals, as also reported by Devi et al. (2023). Nevertheless, variations in nutrient content could be as a result of factors such as fruit variety, nature of cultivating soil and weather conditions.

## Conclusion

It has become necessary to discover ways to improve the nutritional value of our regular diets in order to increase the supply of micronutrients. The results of this study show that the peels contain appreciable quantities of many micronutrients that have been discovered to possess antioxidant activities. Hence, these fruit peels can be processed and used as dietary additives or added to the food production processes to fortify other food items. This study also highlights those peels that have higher concentrations of certain nutrients and these data can be useful in making recommendations in the treatment and/or management of micronutrient deficiencies; mandarin peel, for instance, had an outstandingly higher value of vitamin C. Furthermore, the antinutrient content of the fruit peels was within acceptable limits, which makes them safe for human consumption. The use of fruit peels as dietary additives is encouraged as this will significantly reduce food waste at the household level and in the food industries. Extraction and purification of beneficial antioxidant compounds from the fruit peels are highly recommended.
